# Smoking flies: testing the effect of tobacco cigarettes on heart function of *Drosophila melanogaster*

**DOI:** 10.1242/bio.055004

**Published:** 2021-02-15

**Authors:** Manuela Santalla, Lucía Pagola, Ivana Gómez, Darío Balcazar, Carlos A. Valverde, Paola Ferrero

**Affiliations:** 1 Departamento de Ciencias Básicas y Experimentales, UNNOBA, Monteagudo 2772, Pergamino B2700, Argentina; 2 Centro de Investigaciones Cardiovasculares ‘Dr. Horacio E. Cingolani’, CONICET, Facultad de Ciencias Médicas, Av 60 & 120. UNLP, La Plata B1900, Argentina; 3 Centro de Estudios Parasitológicos y de Vectores, UNLP-CONICET, Bv 120s/n, La Plata B1900, Argentina

**Keywords:** Drosophila, Heart, Tobacco, Downregulation, Nicotine, Receptors

## Abstract

Studies about the relationship between substances consumed by humans and their impact on health, in animal models, have been a challenge due to differences between species in the animal kingdom. However, the homology of certain genes has allowed extrapolation of certain knowledge obtained in animals. *Drosophila melanogaster*, studied for decades, has been widely used as model for human diseases as well as to study responses associated with the consumption of several substances. In the present work we explore the impact of tobacco consumption on a model of ‘smoking flies’. Throughout these experiments, we aim to provide information about the effects of tobacco consumption on cardiac physiology. We assessed intracellular calcium handling, a phenomenon underlying cardiac contraction and relaxation. Flies chronically exposed to tobacco smoke exhibited an increased heart rate and alterations in the dynamics of the transient increase of intracellular calcium in myocardial cells. These effects were also evident under acute exposure to nicotine of the heart, in a semi-intact preparation. Moreover, the alpha 1 and 7 subunits of the nicotinic receptors are involved in the heart response to tobacco and nicotine under chronic (in the intact fly) as well as acute exposure (in the semi-intact preparation). The present data elucidate the implication of the intracellular cardiac pathways affected by nicotine on the heart tissue. Based on the probed genetic and physiological similarity between the fly and human heart, cardiac effects exerted by tobacco smoke in *Drosophila* advances our understanding of the impact of it in the human heart. Additionally, it may also provide information on how nicotine-like substances, e.g. neonicotinoids used as insecticides, affect cardiac function.

This article has an associated First Person interview with the first author of the paper.

## Abstract (precis)

**Summary:** Nicotine, a substance present in tobacco, resembles insecticide compounds such as neonicotinoids, and affects the heart function in *Drosophila melanogaster*.

## INTRODUCTION

*Drosophila melanogaster* (fruit fly) is one of the most extensively studied alternative model for genetics and physiologic studies (Yamaguchi and Yoshida, 2018). The increasing number of transgenic lines that reproduce various aspects of human diseases has been useful for exploring genetic basis of physio-pathological responses to several stimuli, including compounds consumed by humans with relevance to the biomedical field (Heberlein et al., 2009; Santalla et al., 2016; Hong et al., 2018; Gomez et al., 2019). This is possibly due to the similarity between *Drosophila* genes and their orthologous genes in humans.

According to the potentiality of *Drosophila* for exploring human diseases, we decided to study the effect of tobacco cigarettes in the fruit fly. As stated by the World Health Organization (WHO), the tobacco epidemic is one of the biggest public health threats the world has ever faced (WHO, 2020). Death due to tobacco causing serious cardiovascular and respiratory diseases has risen to more than eight million people a year around the world. Interestingly, over seven million of those deaths are associated with direct tobacco consumption, meanwhile about 1.2 million are non-smokers being exposed to second-hand smoke. It is well known that all forms of tobacco are harmful, with no safe level of tobacco exposure (WHO, 2020). Among several consequences of tobacco consumption on health, it has been demonstrated that cigarette smoking increases the risk of atrial and ventricular arrhythmias, sudden death and acute myocardial infarction, and causes hemodynamic changes that exacerbate heart failure (Middlekauff et al., 2014).

Taking advantage of respiration by diffusion or the digestive pathway in *Drosophila melanogaster*, vaporization and/or admixing with meal have been the preferred routes for administrating nicotine. Studies about nicotine exposure were focused on development analysis and larvae or adult sensitivity to this compound. Survival, characteristics of the development stages and adult weight were all affected by nicotine (Velazquez-Ulloa, 2017; Wong, 2017).

The molecular basis of the role of nicotine involves ionotropic nicotinic acetylcholine (ACh) receptors (nAChRs). These receptors are member of a ligand-gated ion channel superfamily present in organisms as diverse as bacteria and humans. They play a function in molecular signalling in neuronal and non-neuronal cells, and they are relevant in neuromuscular junctions (Jones and Sattelle, 2010). The membrane structure of the nAChRs includes five homo or heteromeric subunits arranged around a central cation-permeable pore (Sine and Engel, 2006). Subunits are classified as either alpha or non-alpha according to the presence or absence, respectively, of two adjacent cysteine residues in loop C, relevant for ACh binding (Kao et al., 1984). The specific combination of the subunits determines the functional and pharmacological properties of the receptor. For instance, previous studies on *Drosophila* strains containing a deletion of Dα7 coding gene revealed that Dα7 mediates both developmental and acute effects of nicotine (Velazquez-Ulloa, 2017).

Despite the interest of physio-pathological effects of nicotine in this model, there are few studies about the role of this compound on heart performance. Cholinergic signalling modulates heart rate in many organisms, including *Drosophila*, and therefore nicotine acts as an agonist, as it has been reported in larvae, pupa and adult flies (Zornik et al., 1999; Malloy et al., 2016).

Since in *Drosophila*, as well as in vertebrates, the contraction–relaxation cycle is sustained by a transient increase in calcium concentration within the cytosol of cardiomyocytes, we explored the dynamic of calcium handling in nicotine- and tobacco-exposed flies. The temporary increment in calcium concentration in the cytosolic space in each cycle is known as calcium transient, and changes in its dynamic are reflected as alterations in cardiac mechanical parameters.

In the present study, we hypothesised that repeated exposure to commercial cigarettes modifies the intracellular calcium (Ca^2+^i) handling and that this would conduct to functional changes of the heart. To simulate tobacco consumption, we exposed adult flies to the smoke of commercial tobacco cigarettes by the aid of a custom device, by which flies uptake tobacco smoke as a smoker does. Taking into account the homology of cardiac genes between *Drosophila* and human, we consider that the latter would be an interesting alternative model for delving into the effect of nicotine on heart activity and calcium handling.

Herein, we explore the relevance of the nAChRs in cardiac performance by means of disruption of their integrity downregulating alpha-1 or alpha-7 subunits in the *Drosophila* heart. We demonstrate a differential role of both subunits in the heart function modulation under nicotine and cigarette exposure.

Our contribution to understanding of calcium signalling of the heart under nicotine exposure might be relevant for extrapolating to people who smoke, among other commonly studied effects of tobacco on health. Moreover, there is a family of chemically similar compounds to nicotine that are widely used as insecticides like imidacloprid, thiamethoxam, acetamiprid, dinotefuran and clothianidin (Simon-Delso et al., 2015). There are neurotoxins that act in a similar way that acetylcholine, but their effects in other insects’ cells and organs, like the heart, are not fully studied. In addition, our results contribute to consider the herein described methodology as an inexpensive method for screening of cigarettes with different ratio of compounds as well as synthetic substances in *Drosophila*, and their impact on heart performance.

## RESULTS

**Fig. 1.**
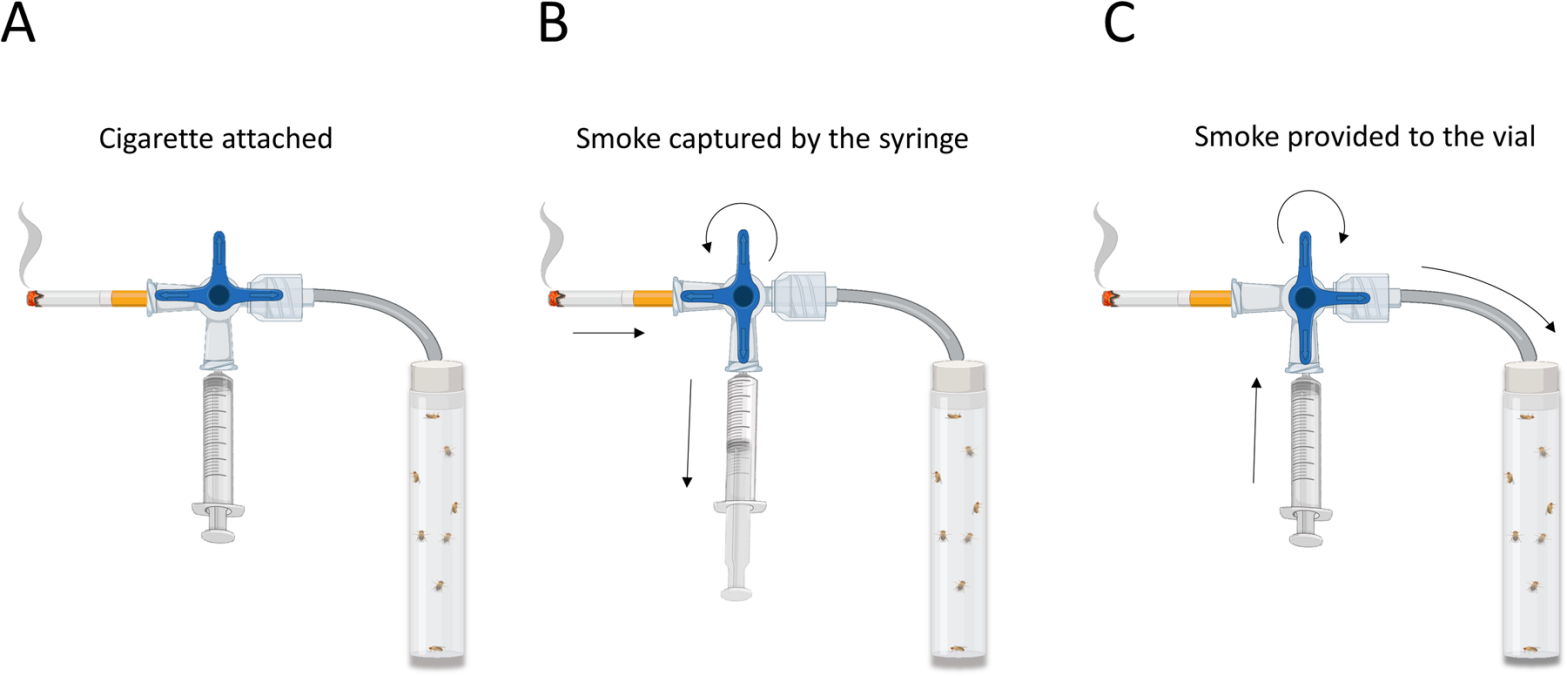
**Device used for tobacco administration to *Drosophila*.** This custom device consists of a tube that contain a three-way stopcock manifold. (A) Device is closed. Cigarette is attached to an extreme of the tube and the three-way stopcock manifold block the hole toward the vial containing flies. (B) The three-way stopcock manifold was rotated and the smoke from the manually burned cigarette is suctioned by a syringe. (C) After loading 10 cm^3^ of smoke, the three-way stopcock manifold is rotated again and the smoke contained in the syringe is sent to a vial with flies, through a tube connected to a vial with flies.

Adult flies housed in a container were exposed to commercial cigarette smoke originated with the aid of a custom device (Fig. 1). The procedure was adapted from a previous study from our group, in which we exposed the flies to vaporized compounds (Gomez et al., 2019). In the present setup, we replaced the vaporizer in the device with a cigarette. By means of a three-way stopcock manifold, smoke was suctioned from the cigarette using a syringe. Afterwards, it was transferred into a vial containing only the flies to be treated. The temperature inside the vial receiving the smoke coming from the cigarette was measured with a probe in order to verify that vial's temperature (room temperature) was not altered during exposition (data not shown). Compounds were up taken through and distributed by the tracheal ramifications of the flies, eventually reaching the hemolymph. Although it is a passive process, we defined it as inhalation. We then analyzed the cardiac performance of *Drosophila* under exposure to cigarette smoke, assessing the parameters associated to the Ca^2+^i handling.

Adult flies (3 days old) were submitted to inhalation of cigarette smoke for 7 days. We tested Phillip Morris^®^ cigarettes, which contain 0.7 mg of nicotine. Two diary doses of 10 cc of smoke were provided and flies were kept in contact with the smoke for 5 min. Subsequently, they were transferred to their vial with food.

After 7 days of smoke exposure, adult flies were euthanized and dissected in order to prepare the semi-intact fly preparation, containing the intact beating heart immersed in artificial hemolymph. This preparation was placed in a confocal microscope in order to assess intracellular calcium transients by means of a genetically-encoded fluorescent reporter system targeted to cardiac cells (Santalla et al., 2014).

**Fig. 2.**
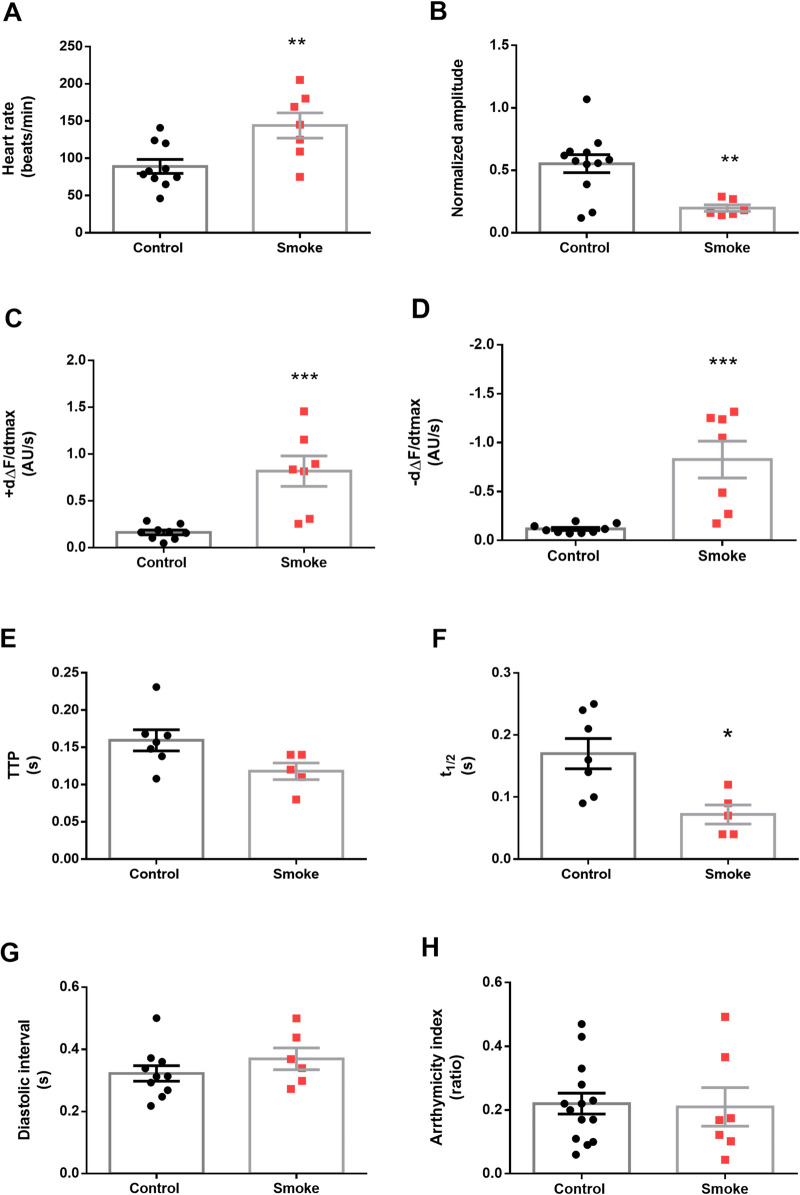
**Commercial cigarette smoke affects cardiac performance of adult *Drosophila*.** Cardiac performance was evaluated in GCaMP3 control flies not exposed (black dots) or exposed to two daily doses of 10 cc of cigarette smoke (red dots) for 7 days. Cigarette smoke exposure increased the heart rate (A) and reduced the Ca^2+^ transient amplitude (B). The maximal velocities of Ca^2+^ transient increase (dΔF/dtmax) and decay (−dΔF/dtmax) were augmented (C,D) in consistency with a shortening of the half-relaxation time (t1/2) (E,F). Diastolic periods and arrhythmia index remained unchanged with treatment (G,H). *N*=4–14 for control and *N*=6–9 for treated flies. All results were expressed as mean± s.e.m. **P<0.01 and ***P<0.001.

Exposure to cigarette smoke incremented the heart rate and reduced the amplitude of the calcium transients (Fig. 2A and B). This was accompanied by an increment of the maximal velocities of contraction and relaxation (+dF/dt, −dF/dt) (Fig. 2C and D). Time to peak (TTP) of contraction did not show a significant decrease although the half-relaxation time (t1/2) showed a faster relaxation (Fig. 2E and F). Diastolic intervals and heart rate variability (AI) did not shown differences (Fig. 2G,H).

**Fig. 3.**
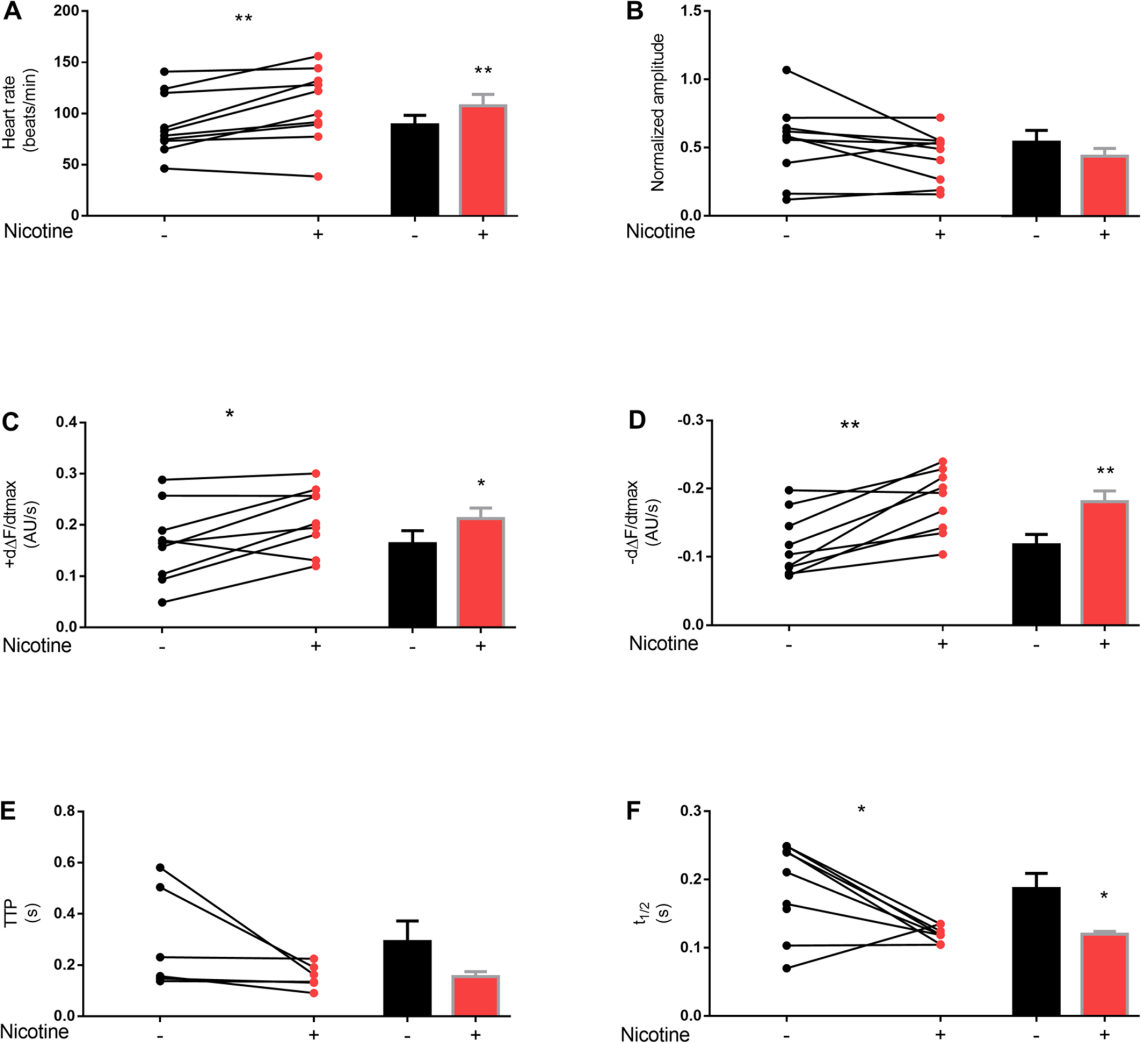
**Acute administration of nicotine mimics some of the cigarette effects.** Intracellular Ca^2+^ cycling parameters of GCaMP3 flies before and after a pulse of nicotine were evaluated. The presence of 7.5 mM of nicotine in the semi-intact preparation incremented the heart rate (A) without changes in Ca^2+^ transient amplitude (B). This was accompanied by an increased maximum rate of Ca^2+^ transient increase (dΔF/dtmax) and decay (−dΔF/dtmax) (C,D). Time to peak of Ca^2+^ amplitude (TTP) did not show significant changes, meanwhile half-relaxation time (t1/2) showed faster calcium transient relaxation (E,F). *N*=6–10 flies. All results were expressed as mean±s.e.m. *P<0.05 and **P<0.01.

In an attempt to elucidate whether nicotine, the relevant component of tobacco cigarettes, is responsible for the observed changes on heart parameters in smoke-exposed flies, we designed a new set of experiments. Nicotine was applied on semi-intact preparation of adult, 7-day-old flies submerged on artificial hemolymph to monitor the activity of the beating heart. Video recording was carried out for a 1 min period. After the first 20 s, nicotine was added to the artificial hemolymph to a final concentration of 1.69×10^−3^ M. The addition of nicotine incremented the heart rate and accelerated the maximal velocities of contraction and relaxation (Fig. 3A,C,D). These results are consistent with the changes observed in flies chronically exposed to cigarette smoke. Although the calcium transient amplitude shows a tendency to decrease, it did not reach statistical significance (Fig. 3B). Similar results were obtained measurement time to peak (TTP) (Fig. 3E). In addition, nicotine-treated flies exhibited a faster half-relaxation time (Fig. 3F). Altogether, these results suggest that nicotine may play a role in cardiac performance under exposure to this substance.

Then, we explored the relevance of the nicotinic acetylcholine receptors (nAChRs) on the effects observed under cigarette exposure. nAChRs are present in vertebrate and invertebrate species. In insects, nAChRs are normally activated by the endogenous ligand, acetylcholine, and these receptors are the site of action of commercially important insecticides such as neonicotinoids (Jones et al., 2007). It has been described that *Drosophila melanogaster* has ten nAChR subunits, namely Dα1-Dα7 and Dβ1-Dβ3 (Lansdell et al., 2012).

**Fig. 4.**
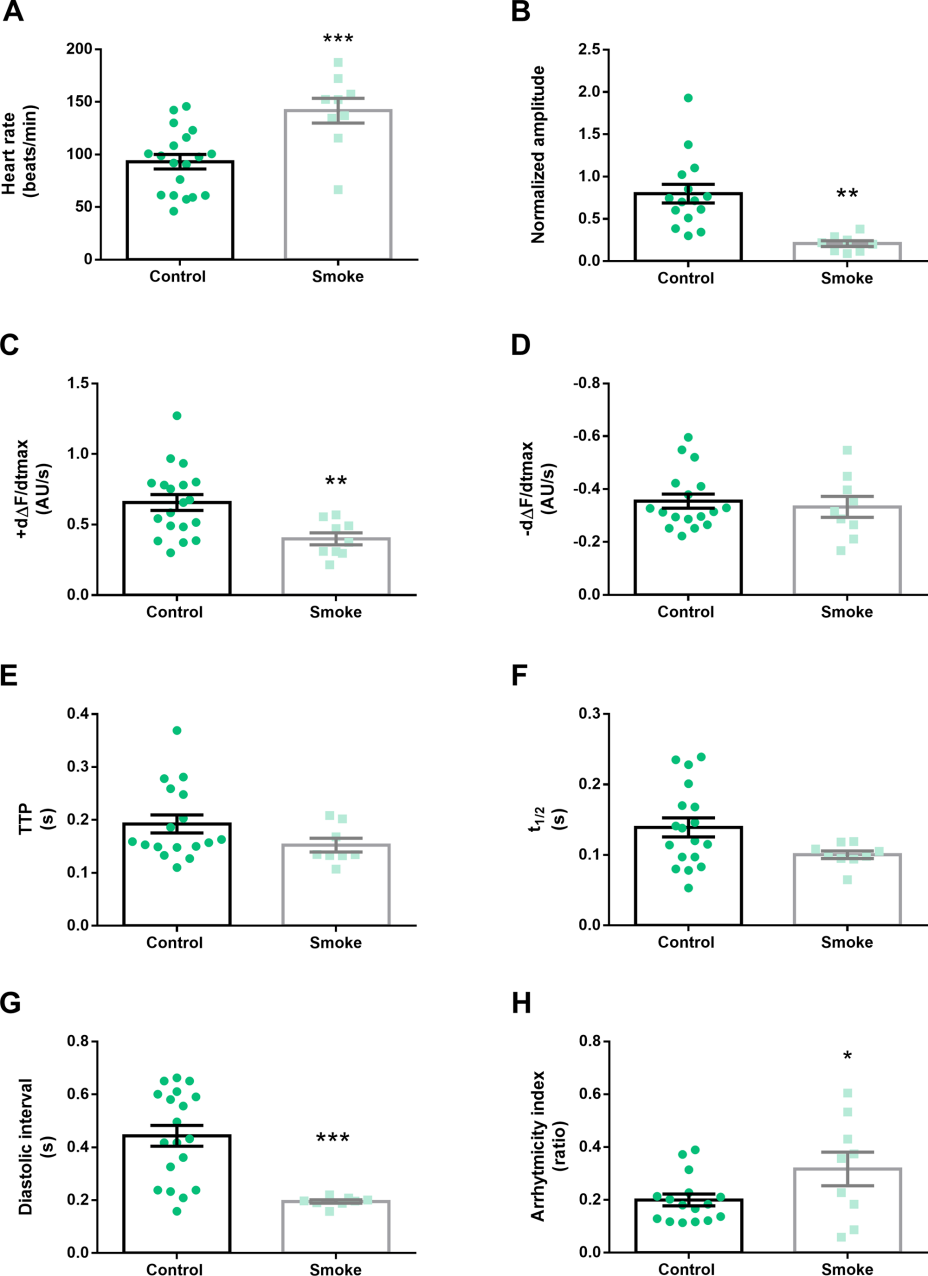
**Downregulation of *Drosophila* nicotinic receptor subunit 1 prevents part of the effects of cigarette smoke.** Intracellular Ca^2+^ cycling parameters were evaluated in flies carrying a cardiac-specific iRNA targeted to the alpha-1 subunit of nAChR. Cigarette smoke increased the heart rate (A) and reduced the Ca^+2^ transient amplitude (B) and the maximum rate of Ca^2+^ transient increase (+dΔF/dtmax) (C) in flies with alpha-1 downregulation. No changes in maximum rate of Ca^2+^ transient decay (-dΔF/dtmax), time to peak (TTP) and half-relaxation time (t1/2) of Ca^2+^ amplitude (D–F) occurred. Smoke reduced the diastolic periods and increased the arrhythmia index in flies harboring the iRNA against alpha-1 subunit (G,H). *N*=15–19 for control and *N*=8–9 for treated flies. All results were expressed as mean±s.e.m. *P<0.05 and **P<0.01.

First, we studied alpha 1 subunit. An interfering microRNA specific for the alpha 1 subunit transcripts was specifically expressed in heart cells, under the tinC driver. Adult flies of the same age were divided into two groups immediately after emerging of the puparium. Flies from one group were exposed to commercial cigarette smoke for 7 days and compared to control group of flies (exposed to ambient air). Downregulation of the alpha 1 subunit of AChRs abolished the impact of smoke on the calcium dynamic, revealed by the absence of change in the maximal velocity of relaxation (-dF/dt), time to peak of contraction and half-relaxation time with respect to the control group (Fig. 4D–F). Unlike wild-type flies, the maximal velocity relaxation (+dF/dt) was reduced (Fig. 4C). However, heart rate was increased and the amplitude of calcium transients was diminished (Fig. 4A,B). Under tobacco smoke exposure, the diastolic interval shows a reduction in concordance with increased heart rate. This was accompanied by a higher variability of heart rate, expressed as arrythmia index (Fig. 4G,H). These results suggest that alpha 1 subunit could be relevant on calcium transient dynamics but without any relevance on heart rate or calcium transient amplitude induced by tobacco cigarettes.

**Fig. 5.**
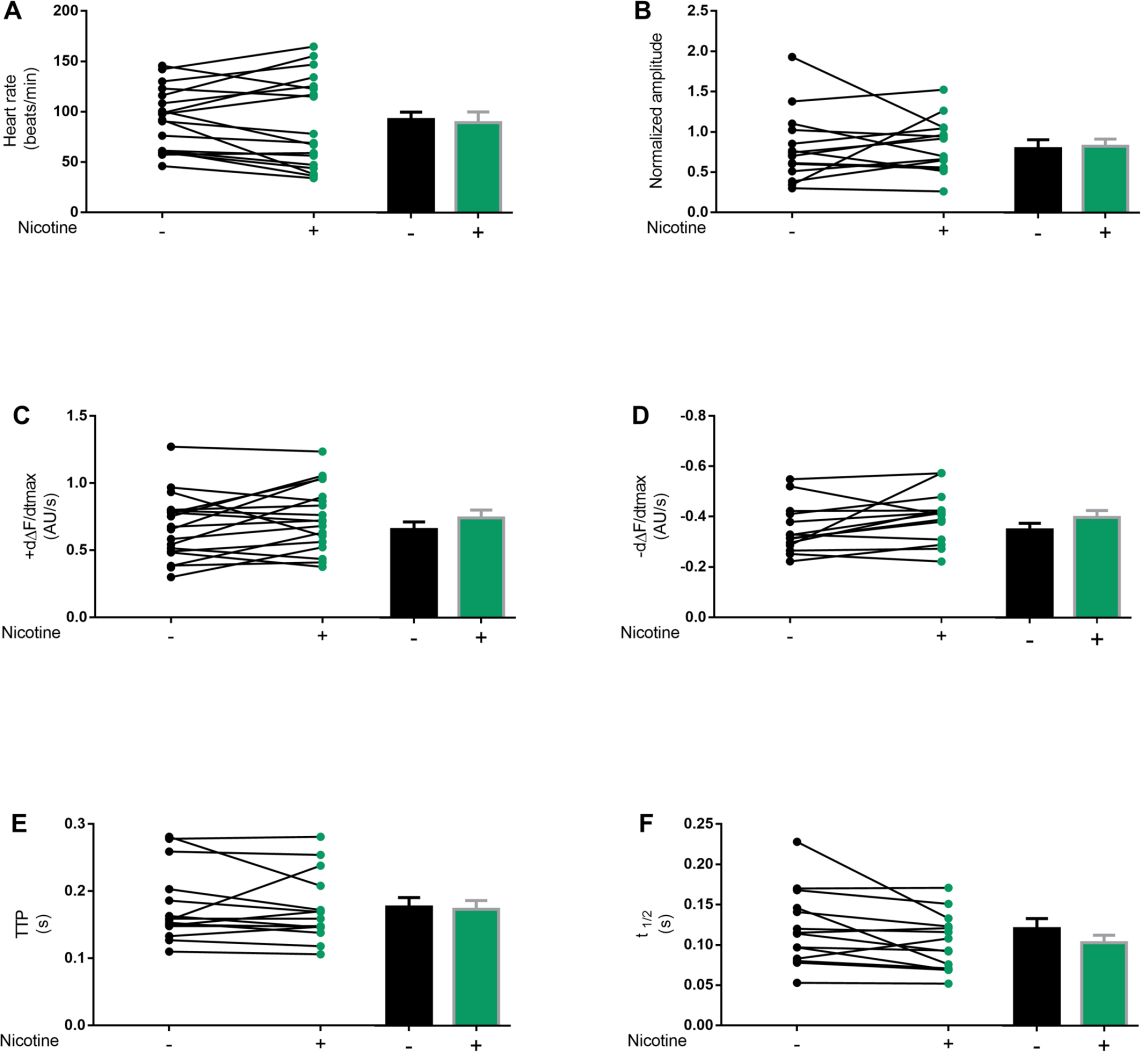
**Cardiac-specific downregulation of alpha 1 subunit of the AChR abolishes the response to nicotine in *Drosophila*’s semi-intact heart preparation.** No changes in heart rate (A), amplitude of Ca^2+^ transient (B), maximum rate of Ca^2+^ transient increase (dΔF/dtmax) and decay (−dΔF/dtmax) and times to peak of Ca^2+^ amplitude (TTP) or to half-relaxation (t1/2) (C-F) were observed in alpha 1-downregulated flies after acute nicotine administration. *N*=14–18 flies. All results were expressed as mean±s.e.m. *P<0.05 and **P<0.01.

Semi-intact preparations of transgenic flies with downregulated alpha 1 gene were not responsive to nicotine exposure (Fig. 5A–F). The increase in heart rate and calcium transient amplitude reduction observed after chronic exposure to cigarette smoke was not evident under acute nicotine challenge (Fig. 5A,B). This would suggest that other components of tobacco cigarette might be affecting the cardiac performance.

The next step was to study the alpha 7 subunit involvement on cardiac performance when fly hearts were exposed to smoke or pure nicotine. The gene encoding the alpha 7 subunits in *Drosophila* (*D***α***7*) is one of the genes with the highest homology to those from vertebrates (Grauso et al., 2002).

**Fig. 6.**
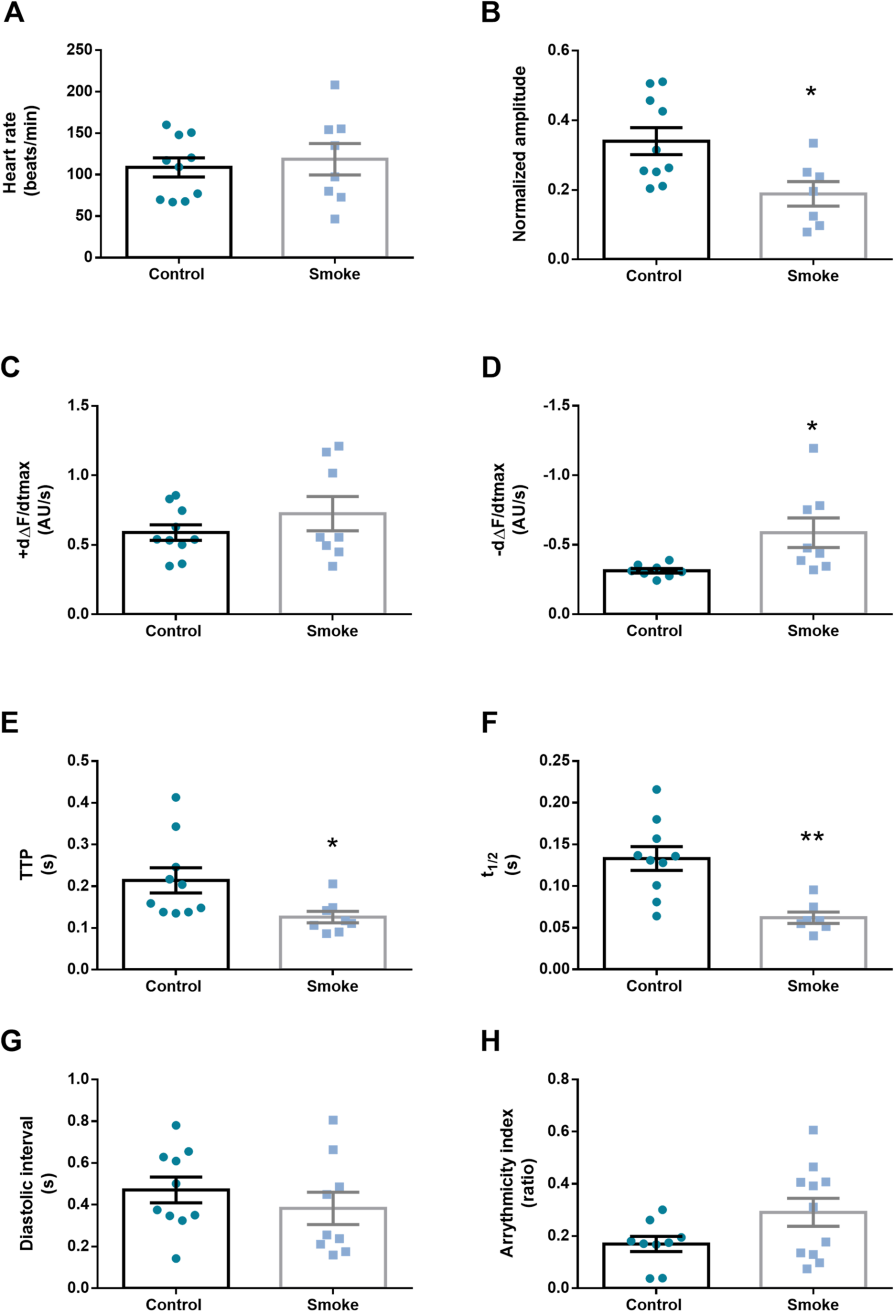
**Downregulation of *Drosophila* nicotinic receptor subunit 7 prevents part of the effects of cigarette smoke.** Flies expressing the iRNA targeted to the alpha 7 subunit of nAChR in cardiac tissue were exposed to tobacco smoke arising from a commercial cigarette, in comparison to non-exposed flies. No changes in heart rate (A) and maximum rate of Ca^2+^ transient increase (dΔF/dtmax) (C) were observed. Amplitude of Ca2+ transient showed and increase (B) in flies which were exposed to cigarette smoke. Increase of maximum rate of decay (−dΔF/dtmax) (D), shortening of TTP and t1/2 (E,F) were observed. Diastolic periods and arrhythmia index and remained unchanged with treatment (G,H). *N*=8–10 for control and *N*=7–11 for treated flies. All results were expressed as mean±s.e.m. *P<0.05 and **P<0.01.

In contrast to wild-type and alpha 1-downregulated *Drosophila melanogaster*, flies carrying the iRNA (expressed under the cardiac-specific promotor TinC) targeted to the alpha-7 subunit of AChR did not exhibit increased heart rate when exposed to cigarette smoke (Fig. 6A). Similar results were obtained for the diastolic intervals and AI (Fig. 6G,H). However, most of the analyzed parameters, i.e. the calcium transient, maximal velocity of relaxation, time to peak and time to half relaxation showed significant changes when flies were exposed to cigarette smoke respect to control flies (Fig. 6B–F). These results suggest that alpha 7 subunits are not essential mediators of cigarette impact on these cardiac calcium transient parameters regulation.

**Fig. 7.**
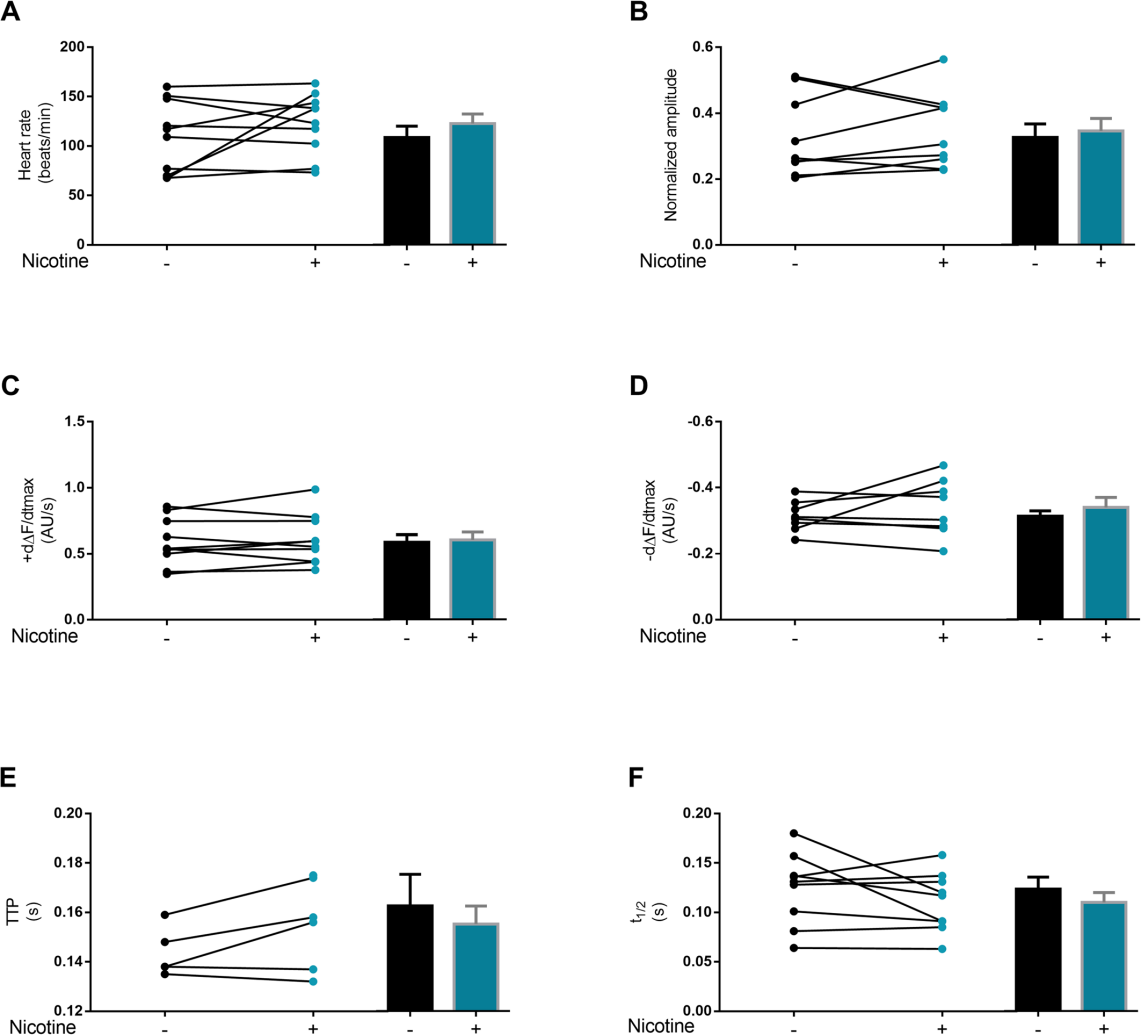
**Cardiac-specific downregulation of alpha 1 subunit of the AChR abolishes the response to nicotine in *Drosophila*’s semi-intact heart preparation.** No changes in heart rate (A), amplitude of Ca^2+^ transient (B), maximum rate of Ca^2+^ transient increase (dΔF/dtmax) and decay (−dΔF/dtmax) and times to peak of Ca^2+^ amplitude (TTP) or to half- relaxation (t1/2) (C–F) were observed in alpha 7-downregulated flies after acute nicotine administration. *N*=5–10 flies. All results were expressed as mean±s.e.m. *P<0.05 and **P<0.01.

Another set of experiments testing the action of nicotine in these alpha 7-downregulated transgenic flies, showed that cardiac parameters are not significantly different respect to the control group (Fig. 7A–F). Therefore, alpha 7 subunit would be part of the underlying mechanism that mediates the response to nicotine exposure but no to other components present in tobacco cigarettes.

In summary, alpha 1 and 7 subunits would participate in the nicotine-associated effects on cardiac performance. Interestingly, alpha 1 subunit, but not alpha 7, would play a role associated to response to non-nicotine components of tobacco cigarettes.

Our results demonstrate that cardiac specific expression of microRNA targeted to alpha 1 or alpha 7 subunits of nAChRs modifies the response to smoke exposure from commercial cigarettes and abolishes the response to nicotine in semi-intact preparation of fly's heart. This suggests that both subunits are part of receptor structures which might be present in nervous terminal and/or in cardiac tissue, modulating the effect of nicotine on cardiac performance.

## DISCUSSION

Administration of chemical substances in an experimental model as *Drosophila* adult hearts allows assessment of several relevant aspects: (1) the response to various compounds, monitoring behavioural changes, neural activity, reaction to stimuli, among others; (2) dose-scaling studies and routes of administration, by admixing the substances with water, food, or dissolving them in an appropriate vehicle to be vaporized for acute or chronic administration; (3) analysis of different forms of a substance, e.g. pure, combined or in the context of the natural origin in which is usually present; (4) the response in mutant flies, which provides insights about the mechanism of action of the tested substances; and (5) the feasible extrapolation of this information to vertebrates, including human, based on the many human orthologue genes with *Drosophila*.

In this work, we tested tobacco commercial cigarettes in *Drosophila* hearts. We focused on the impact of tobacco, particularly nicotine, on the cardiac activity. Flies exposed to tobacco cigarettes for 7 days showed changes on cardiac function. These results were fully reproduced by application of an acute pulse of nicotine in semi-intact preparation containing only the isolated heart with minimal nervous innervation (Dulcis and Levine, 2005; Vogler and Ocorr, 2009), indicating that nicotine has a direct effect on heart performance.

Regarding the role of nicotine on heart activity, it has been described that application of nicotine induces a decrease in the heart rate of larvae, pupa and adult *D. melanogaster* when nicotine (10^–^^4^ M, 10^−3^ M, or 10^−2^ M doses) is injected into the animal (Zornik et al., 1999). Malloy et al., (2016) shows that larval heart rate increases but when exposed to lower concentrations (10^−7^ to 10^−3^ M). Our results exhibited an increased heart rate in adult flies subjected to a pulse of 1.69×10^−3^ M nicotine. This concentration was selected in accordance to the range of nicotine concentration used by Zornick et al., for the adult stage of *Drosophila* (Zornik et al., 1999). However, the different response to this stimulus in our experiments when compared to Zornik et al., might be due to the different route of administration, heart rate recording *in vivo* versus semi-intact preparation, and time between nicotine application and assessment of the heart rate. Summarizing, different stages of development, nicotine doses, route of administration, as well as the exposition period, can be at the basis of the apparent differences. For chronic treatment, doses applied were determined by the nicotine concentration in the cigarette. We are aware that further experiments are required to compare levels of these substances and their metabolites under chronic exposure in animal models. In humans, measurements on serum are based in cotinine (nicotine metabolite) levels and total nicotine equivalents (TNE) which is the sum of urinary nicotine, cotinine and several metabolites in the nicotine metabolic profile (Chang et al., 2017). In our conditions, added to increased heart rate, acute and chronic exposure to nicotine increased the rate of contraction and relaxation of the calcium transient.

Afterward, we explored the nicotinic acetylcholine receptors (nAChRs) as possible mediators of the effects observed under nicotine exposure in semi-intact preparation, as well as under chronic exposure to smoke obtained from commercial cigarettes. nAChRs mediate the fast actions of acetylcholine at cholinergic synapses (Jones et al., 2007). In vertebrates, nAChRs play a significant role in modulating glutamatergic, GABAergic, and dopaminergic neurotransmission (Feduccia et al., 2012). For instance, different glutamatergic receptors are critically involved in several nicotine-mediated effects (Marchi et al., 2015). In *Drosophila*, acetylcholine (Zornik et al., 1999; Malloy et al., 2016), serotonin (Majeed et al., 2014), dopamine (Zornik et al., 1999; Titlow et al., 2013), glutamate (Dulcis and Levine, 2005), octopamine (Zornik et al., 1999; Sujkowski et al., 2020) and melatonin (VanKirk et al., 2017) are capable of regulating the cardiac performance. Activation of cholinergic, dopaminergic and serotonergic neurons stimulates the release of cardioactive substances that increase heart rate in *Drosophila* (Malloy et al., 2017). Semi-intact preparation is deprived of innervation and acute pulse of nicotine on heart seems to be mediated by a direct effect on cardiac tissue. However, in chronic exposure to tobacco, the central nervous system might play a role on nicotine-mediated effects on heart performance, considering that the *Drosophila* adult heart is innervated by glutamatergic neurons (Dulcis and Levine, 2005).

In acute as in long-term exposure to cigarette smoke, either in active or passive smokers, nicotine effects are associated with a sympathetic activation. For instance, in humans, those effects involve acute increase of blood pressure and heart rate, which are potentially dangerous for patients with heart failure, coronary artery disease and arrhythmias (Middlekauff et al., 2014). *Drosophila* possess a counterpart of the adrenergic system in vertebrates, the octopaminergic system, in which octopamine is functionally comparable to norepinephrine that modulates heart performance (Sujkowski et al., 2020). In our conditions, in chronic exposure to tobacco, this signalling pathway might influence on heart activity and we believe that it should be explored in the future.

Herein, focusing in AchRs, transgenic flies with cardiac specific alpha 1 downregulation exhibited altered relaxation after chronic exposure with respect to control flies. These flies with reduced expression of alpha 1 subunit were not responsive when there were subjected to a pulse of nicotine. On the other hand, cardiac specific downregulation of alpha 7 subunit turned the flies unresponsive to tobacco-induced increment on heart rate and reduced calcium transient amplitude. However, direct administration of nicotine to the semi-intact preparation did not modify any parameter, as for alpha 1-interfered strain.

Both alpha 1 and alpha 7 downregulation abrogates the effects of direct application of nicotine to the heart. Nevertheless, downregulation of either subunits (alpha 1 or 7) exhibited a different impact on the cardiac effects possibly mediated by an extra component(s) present in tobacco cigarettes. This behaviour should be explored more deeply in the future. One aspect that must be considered is the particular combination of subunits in homo or heterodimers, which determines the functional and pharmacological properties of nAChRs.

ACh receptors are ubiquitous in the CNS of *Drosophila*, but their expression in cardiac tissue still needs to be fully determined. We did not determine the expression level of nAChR protein or transcript in heart tissue. In our hands, the results with alpha 1 and alpha 7 interference specifically targeted to the cardiac cells suggest that nAChRs mediate the effects on heart parameters induced by nicotine and maybe additional cigarette compounds. nAChRs might be present in the neuronal terminals that innervate the cardiac muscle, although it has been described that only glutamatergic neurons and peripheral crustacean cardioactive peptide neurons reach the *Drosophila* heart (Dulcis and Levine, 2005). This is an issue that requires to be explored more deeply in future studies.

There is evidence supporting dose-dependent response to nicotine, although desensitization of receptors exposed to high doses is relevant. For instance, flies reared on food containing 0.3 mg/ml nicotine throughout development or during the larval stages showed a decreased sensitivity to acute nicotine administration on a negative geotaxis test (Velazquez-Ulloa, 2017). *In vitro* analysis in dissociated central nervous system neurons from insects suggested desensitization of nAChRs (Wegener et al., 2004; Thany et al., 2007). Malloy et al. found that larval locomotion was reduced in association with an increment in the dose (0.001 M, 0.01 M and 0.1 M) and time (20 min or 24 h of duration) of exposure to nicotine (Malloy et al., 2019). nAChRs desensitization was described as the probably underlying phenomenon associated with this response (Malloy et al., 2019). In our assays, nicotine was applied for 30–40 s, as an acute exposure to the compound, considered as a short enough exposition-time to induce nAchRs desensitization. Moreover, nicotine is a lipophilic compound, capable to cross the plasma membrane at pH 7.4 (normal pH of the perfusion solution) (Hukkanen et al., 2005). Once inside the cell, it has been reported that nicotine binds to nascent nAChRs and acts as a stabilizing pharmacological chaperone, favouring the endoplasmic reticulum exit of nAChRs (Kuryatov et al., 2005; Sallette et al., 2005; Lester et al., 2009). This mechanism contributes to the upregulation of nAChRs on the plasma membrane (Shivange et al., 2019). In neuronal tissue, it has been described that nicotine leads to increase in intracellular Ca^2+^ in pre-synaptic terminals due to an increment on calcium conductance of nAChR subtypes, and indirectly via intracellular signalling cascades (Zhong et al., 2015). Various combination conforming the nAChRs exhibit similar pharmacology but differ in their kinetics and sensitivity to nicotinic agonists and antagonists (Dupuis et al., 2012). This issue might be worthy to be explored in the future, testing the behaviour of different nAChRs subtypes and the nicotine induced heart response in *Drosophila*.

In a wider context, far beyond cardiac activity, the nicotine sensitivity of the fly is manifested by other changes, e.g. acute exposure of adult flies to volatilized nicotine impairs their ability to climb in a negative geotaxis assay (Bainton et al., 2000). It has been reported that adult *Drosophila* lose their negative geotaxis reflex for a period of time that is proportional to the amount of the vaporized nicotine administrated (Sanchez-Diaz et al., 2015). Nicotine could be harmful in healthy individuals although it might play a beneficial role in diseases. In the brain, nicotine enhances vesicular release of dopamine. Dopamine depletion is a hallmark of Parkinson’s disease. Studies carried out in *Drosophila* demonstrates that nicotine improved survival of paraquat-treated flies, used as a Parkinson’s disease model, and modifies the expression patterns of particular genes as CG14691, one homologous gene of the human SV2C which encode a synaptic vesicle protein involved in the release of dopamine (Hill-Burns et al., 2013). Moreover, has been demonstrated that chronic nicotine exposure improved viability and flying capability in other models of Parkinson’s disease (Chambers et al., 2013).

It has been also noted that nicotine exposure delayed development and decreased adult sensitivity to nicotine and ethanol. The *Drosophila* alpha *7* subunit mediates nicotine-induced effects on survival, developmental delay, and it may also have a role on nicotine-induced sensitivity to acute nicotine administration (Velazquez-Ulloa, 2017). Perry et al. have identified mutations in the α and β subunits of *Drosophila*’s nAChR that confer reduced sensitivity to several neonicotinoids, a widely used new class of insecticides that act on postsynaptic nicotinic receptors (Perry et al., 2008).

Herein, we described a novel approach for assessing the effects of certain substances (e.g. nicotine) in adult flies; and moreover, we assess the cardiac performance under exposure to tobacco smoke or nicotine. This kind of experiments are useful in two aspects. First, they allow to examine the response of insects to these compounds, which are present in a new class of insecticides. Secondly, this methodology allows exploration of orthologous genes of alpha 1 and alpha 7 subunits of nAChRc in vertebrates and even in humans. The studies in an alternative model as *Drosophila* contribute to decipher the underlying mechanisms and genetic basis of the responses to substances and the exploration of several disease models.

## MATERIALS AND METHODS

### Drosophila strains

All fly stocks were housed, grown and bred at 25°C on standard cornmeal-yeast medium.

To evaluate cardiac calcium handling a strain carrying the heart-specific reporter system *TinC- Gal4-UAS-GCaMP3* (called GCaMP3) was used (Lin et al., 2011).

Transgenic flies that expressed iRNA targeted to the alpha 1 and 7 subunits of the nicotine receptor under UAS sequence (BDSC_28688 and BDSC_51049) (Perkins et al., 2015) were crossed with flies harbouring the reporter system UAS-GCaMP3 and the Gal4 activator under the control of heart-specific promoter, *tinC*. Heterozygous individuals of F1 progeny were grown at 25°C. Progeny resulting from Canton-S crossed with flies harbouring the reporter system was used as control in order to evaluate the iRNA effects in the same genetic background of the reporter system.

### Commercial cigarette smoke administration

By means of a homemade device (Gomez et al., 2019), two doses of 10 cc of commercial cigarette smoke (composition in mg for each cigarette: 10 tar, 0.7 nicotine and 10 carbon monoxide) were administered daily for 7 days starting on day 3 after hatching. Flies were exposed to the tobacco smoke for 5 min each time at an interval of 6 h. Afterwards, the flies were dissected and hearts were used for assessing intracellular calcium handling.

### Acute administration assays: pulse of nicotine

In order to assess if one of the main components of commercial cigarettes, nicotine, was responsible for the effects associated with the administration of tobacco smoke, nicotine was added to the hemolymph (final concentration of 1.69 mM in the preparation).

Semi-intact preparation was mounted in a confocal microscope (Carl Zeiss 410) and the fluorescent signal of the calcium sensitive reporter was recorded for 30 s. Next, nicotine was added to the preparation and calcium signal was immediately recorded for the following 30 s.

### Heart dissection

Hearts were dissected and prepared as described by Santalla et al. (2014). Briefly, adult flies were anaesthetized with CO2, and thorax and legs were removed from the fly. Flies were bathed in oxygenated hemolymph buffer (in mM, 108 NaCl, 5 KCl, 8 MgCl2, 1 NaH2PO4, 4 NaHCO3, 5 HEPES, pH 7.1, 10 sucrose, 5 trehalose and 2 CaCl2) (Vogler and Ocorr, 2009; Santalla et al., 2014; Balcazar et al., 2018) at room temperature. After dissection, semi-intact preparations of adult hearts were maintained for 40–45 min. In cases in which this procedure might take longer time for analysis, other hemolymph preparations have been shown to be useful to preserve the physiological states of the organ (de Castro et al., 2014, 2019).

### Functional analysis

Beating hearts from semi-intact preparations were observed under a confocal microscope (Carl Zeiss 410), and changes in GCaMP3 fluorescence in the first cardiac chamber (conical chamber) were recorded. Transient elevation of cytosolic Ca^2+^ concentration that precedes cardiac contraction was detected as a fluorescent signal. Measurements included: Ca^2+^ transient amplitude (ΔF/F0, expressed in arbitrary units of fluorescence, AU), maximal rates of increasing and decreasing fluorescence (+dΔF/F0/dt) (in AU/second), (−dΔF/F0/dt) (in AU/second), time to peak of Ca^+2^ transient (TTP) (seconds) and half-relaxation time t1/2 (seconds). Number of fluorescent events per minute were counted to obtain heart rate, and the diastolic periods (stable fluorescent signal between calcium transients) were measured. Arrhythmicity index (AI) was calculated as the standard deviation of periods (time between maximal fluorescence of consecutive beats) normalised by their average (Balcazar et al., 2018).

### Statistical analysis

All results from functional analyses of calcium handling were expressed as mean±s.e.m. Statistical analysis was made by comparisons using Student’s *t*-test paired for acute assays (application of nicotine pulse to the fly preparation). Unpaired Student’s *t*-test was used for comparisons of the effects of commercial cigarette among different individuals. *P*-values<0.05 were considered statistically significant.
